# Editorial: Biochemical biomarkers of nutritional status

**DOI:** 10.3389/fnut.2024.1397071

**Published:** 2024-04-12

**Authors:** Cheng Zheng, Elad Tako

**Affiliations:** ^1^Department of Biostatistics, University of Nebraska Medical Center, Omaha, NE, United States; ^2^Department of Food Science, Cornell University, Ithaca, NY, United States

**Keywords:** nutritional status, biomarker, nutrients, diet, biochemical

Diet plays an important role in health maintenance and prevention of chronic diseases. Nutritional status involves the balance between nutrient intake, absorption, and utilization, and for several reasons may become unbalanced due to deficits or excesses in any of these domains. A nutritional biomarker can be defined as a short- or long-term biochemical indicator of nutritional status and/or dietary intake, related to constituents of nutrient metabolism or determining the biological effects of dietary intake. Objectively measured biochemical biomarkers of nutrient intake using serum, plasma, urine, and stool specimens are needed to establish reliable diet-disease associations.

Biochemical biomarkers of nutrient circulation and storage have long been used in clinical practice but have limitations especially in the setting of acute and chronic diseases. The accurate assessment of such nutrient intake is challenging, and robust biomarkers of nutrient intake are scarce. In this sense, there is a need for accurate and reliable analytical determinants that can accurately reflect the individual's nutritional status, exposure and biological effects (bioavailability) of nutrients.

With the potential high dimensionality of the measurements from different specimens from different resources, new methods are needed for new biomarker development and its application in diet-disease association studies. Biomarkers are tools of great importance for nutrition, both in terms of nutritional diagnosis and in helping to validate methods for assessing food intake. In addition, with advances in omics science studies, new promising biomarkers may contribute even more to this area.

This Research Topic aims to collate original research and systematic review articles to highlight recent advances in the development, validation, and implementation of “*Biochemical biomarkers of nutritional status*” in health and disease situations, specifically biochemical biomarkers of nutrient intake and their bioavailability.

In this Research Topic, there are six distinct papers showcasing the breadth and depth of contemporary nutrition research related to above Research Topics.

Liu et al. present a case report which uncovers the diagnosis of an asymptomatic mother with inborn errors of cobalamin metabolism (cblC) using high plasma and urine homocysteine levels during prenatal diagnosis. This work highlights the importance of early detection and management of poor nutritional status with a biochemical biomarker.

Jones et al. showcase a study employing liquid-chromatography tandem mass spectrometry (LC–MS/MS) as a robust method for quantifying vitamin D concentrations in human milk, shedding light on its nutritional significance for infant health. The work showed that a good biomarker is essential to inform public health policies around vitamin D fortification and supplementation.

Shi et al. reveal a novel nutritional index associated with stroke risk in the Chinese population with hypertension, offering potential implications for preventive strategies.

Nakamura et al. explores the ketogenic effects of medium chain triglycerides-containing formula, correlating with breath acetone levels in healthy volunteers. This research contributes to understanding the metabolic effects of dietary interventions.

Li et al. investigates the prevalence and prognostic implications of malnutrition in early-stage multiple system atrophy, underscoring the importance of nutritional assessment and intervention in neurodegenerative disorders.

Zhang et al. propose a novel general approach utilizing a controlled feeding study and collected high-dimensional serum and urine metabolites in such a study to develop objective nutritional biomarkers. Such biomarkers are further used for correcting the measurement error in self-reported data via a regression calibration approach. The work showcases an advanced statistical method developed for nutritional biomarker discovery and the evaluation of nutrition-disease relationships with such biomarkers.

Together, these papers exemplify the diverse frontiers of nutrition research, spanning from genetic disorders to metabolic pathways, nutritional epidemiology, and neurodegenerative diseases. They underscore the interdisciplinary nature of modern healthcare and offer valuable insights for improving diagnostics, treatment strategies, and preventive measures across various medical domains.

## Author contributions

CZ: Writing – original draft, Writing – review & editing. ET: Writing – review & editing.

